# The Effect of Bacterial Infection on the Biomechanical Properties of Biological Mesh in a Rat Model

**DOI:** 10.1371/journal.pone.0021228

**Published:** 2011-06-16

**Authors:** Charles F. Bellows, Benjamin M. Wheatley, Krzysztof Moroz, Stephanie C. Rosales, Lisa A. Morici

**Affiliations:** 1 Department of Surgery, Tulane University Health Sciences Center, New Orleans, Louisiana, United States of America; 2 Department of Pathology, Tulane University Health Sciences Center, New Orleans, Louisiana, United States of America; 3 Department of Microbiology and Immunology, Tulane University Health Sciences Center, New Orleans, Louisiana, United States of America; Université de Technologie de Compiègne, France

## Abstract

**Background:**

The use of biologic mesh to repair abdominal wall defects in contaminated surgical fields is becoming the standard of practice. However, failure rates and infections of these materials persist clinically. The purpose of this study was to determine the mechanical properties of biologic mesh in response to a bacterial encounter.

**Methods:**

A rat model of *Staphylococcus aureus* colonization and infection of subcutaneously implanted biologic mesh was used. Samples of biologic meshes (acellular human dermis (ADM) and porcine small intestine submucosa (SIS)) were inoculated with various concentrations of methicillin-resistant *Staphylococcus aureus* [10^5^, 10^9^ colony-forming units] or saline (control) prior to wound closure (n = 6 per group). After 10 or 20 days, meshes were explanted, and cultured for bacteria. Histological changes and bacterial recovery together with biomechanical properties were assessed. Data were compared using a 1-way ANOVA or a Mann-Whitney test, with p<0.05.

**Results:**

The overall rate of staphylococcal mesh colonization was 81% and was comparable in the ADM and SIS groups. Initially (day 0) both biologic meshes had similar biomechanical properties. However after implantation, the SIS control material was significantly weaker than ADM at 20 days (*p* = 0.03), but their corresponding modulus of elasticity were similar at this time point (p>0.05). After inoculation with MRSA, a time, dose and material dependent decrease in the ultimate tensile strength and modulus of elasticity of SIS and ADM were noted compared to control values.

**Conclusion:**

The biomechanical properties of biologic mesh significantly decline after colonization with MRSA. Surgeons selecting a repair material should be aware of its biomechanical fate relative to other biologic materials when placed in a contaminated environment.

## Introduction

Incisional hernias are one of the most common complications following abdominal surgery. The use of implantable synthetic mesh material has proven to be the preferred method of hernia repair to decrease the recurrence rates [1]. But this lower recurrence rate comes at a price of mesh-related complications such as extrusion, chronic pain, entero-cutaneous fistula formation and infection [2]–[4].

To avoid the potential sequelae of synthetic prosthetic mesh, biological prosthetics have been developed and used for hernia repair. These materials are all essentially composed of an extracellular matrix (ECM) stripped of its cellular components, but differ substantially in their source (porcine small intestine submucosa, porcine dermis or cadaveric human dermis), de-cellularization and sterilization methods [5]. The ECM represents nature's ideal biologic scaffold and an excellent substrate for cell attachment, proliferation, and differentiation [5], [6]. Interestingly, the literature makes claims that these biologic meshes are also “resistant” to infection, yet clinical studies have shown that infection continues to complicate the use of these materials [7]–[11].

The ability of these materials to resist the influence of bacterial persistence on the implantation site is most likely a function of the bacteria communities, the composition of the biologic mesh and the morphologic properties of its surfaces, as well as the interaction with the host's local tissue defenses. Some researchers believe that an insidious perpetual fight between invading pathogens and the patient's immune system turns the surgical site to an inflamed battleground, resulting in a constant release of inflammatory mediators which subsequently end in mesh degradation and significant loss of function and finally recurrence of the abdominal wall hernia. It is possible that no biological mesh could hope to withstand an overwhelming infection. However, to date, no investigators have addressed the effect of a bacterial colonization on the biomechanical properties of these biologic meshes *in vivo*. This interaction is an important as well as interesting field for research, since infection is the most important complication following implantation of a surgical mesh. Therefore, the purpose of this study is to compare the influence of a bacterial encounter on the biomechanical stability of two different, commonly used, biologic prostheses *in vivo*. This information may help surgeons in determining which biologic mesh is acceptable to implant into a contaminated or potentially contaminated surgical field.

## Materials and Methods

### Ethics Statement

This study was approved and monitored by Tulane University's Institutional Animal Care and Use Committee, and all animals were cared for in accordance with guidelines of the Association for Assessment and Accreditation of Laboratory Animal Care International (#4026R).

### Bacterial Inoculum Preparation

The bacterial strain, methicillin-resistant *Staphylococcus aureus* (MRSA), was obtained from American Type Culture Collection (ATCC #43300). One day prior to implant, an aliquot of this strain was thawed from frozen stock and grown overnight in Luria-Bertani broth (LB) for 16–19 hours. The overnight cultures was diluted 1:100 into fresh LB and grown for 3 hours. The cultures were washed in saline, and the culture concentration was determined by spectrophotometry (OD_600_) and compared to a predetermined growth curve. Each culture was brought to the desired concentration and verified by plating dilutions of the final solution.

### Experimental Animals and Design

Male Sprague Dawley rats (Charles River Laboratories International, Inc., Wilmington, MA), each ranging from 300 to 500 grams had dorsal subcutaneous pockets created using an established model, described briefly below. The animals were randomly assigned to receive one of the two FDA- approved biologic meshes, for hernia repair, commonly used in clinical practice acellular dermal matrix (ADM; AlloDerm; Life Cell, Branchburg, NJ) or multi-laminate (8-layer) porcine small intestinal submucosa (SIS; Surgsis Biodesign; Cook Biotech, Bloomington, IN). Rectangular implants (2.5×1.5 cm) were fashioned from each material using a sterile plastic template, and rehydrated in sterile saline immediately before implantation. The control (non-infected) animals (*n* = 6) received a piece of rehydrated ADM or SIS and 200 µL of sterile normal (0.9%) saline into the surgical wound. The experimental (infected) animals received a piece of rehydrated ADM or SIS inoculated with a 200 µL bacterial suspension of 10^5^ cfu or 10^9^ cfu (colony forming units) MRSA into the surgical wound before skin closure. All animals were then monitored for 10 or 20 days and then sacrificed. Repair sites were explored, clinical observations were made, and the mesh excised under sterile conditions for histologic, microbiologic, and mechanical analyses, as described below.

### Mesh Infection Rat Model: Implantation Procedure

All animals were anesthetized using inhaled isoflurane initially via induction chamber and maintained by a nosecone (1–5% oxygen). The back of each rat was then clipped and cleaned with povidone and allowed to dry for 2 minutes. According to the model by Darouiche and colleagues, the biologic mesh implants were placed under dorsal skin flaps [12]. Briefly, using sterile techniques bilateral dorsal incisions were made 1 cm to each side of the spine measuring 3 cm in length. Using blunt dissection, a subcutaneous pocket was developed at each incision site. The tissue dissection was meticulously done to create the smallest-size pocket necessary to accommodate each mesh and to provide adequate hemostasis. Once the pocket was created, one piece of re-hydrated (for 30 minutes) mesh was placed in each subcutaneous pocket. The bacterial inoculum or saline was then pipetted onto the top of the implanted mesh. All wounds were then closed using stainless steel skin clips (EZ Clips, Braintree Scientific, Inc). After skin closure, Buprenorphine (0.02–0.05 mg/kg) was administered intramuscularly every 12 hours for 3 days. After achieving sternal recumbence, the rats were housed individually and left for either ten or twenty days with available food and water. The animals were monitored daily for signs of pain, distress, erythema, local infection and sepsis. Incisions were observed to detect macroscopic findings of infection such as seroma formation, wound dehiscence, and purulent drainage. Skin clips were typically removed 7 days postoperatively.

### Harvest: Collection of Samples

At ten and twenty days postoperatively, the animals were anesthetized, and a careful dissection was performed to open the dorsal flap. The underlying implant was evaluated carefully and excised, if needed, with surrounding tissue using sterile instruments. One implant per animal was placed in a Petri dish containing 2 ml of 0.9% saline to remain hydrated prior to biomechanical analysis. The other implant was cutinto two equal pieces. One piece was fixed for 24 h in 10% buffered formalin (Fisher Scientific, Fair Lawn, NJ) and processed according to conventional procedures for histologic assessment and the other piece was placed in a tube containing 1 ml sterile saline and immediately analyzed by serial dilution plating for any bacteria present.

A cardiac puncture was then taken after which the animal was humanely euthanized. This blood drawn at study termination was used to determine if there was any hematogenous dissemination of the bacteria which could lead to multi-device colony counts in the same animal. A 100 µl aliquot of whole blood was inoculated on a blood agar plate. Bacterial growth was assessed after the plates were incubated at 37°C for forty-eight hours.

### Bacterial recovery at explant

Several initial *in vitro* bacterial sampling methods, on both biomaterials, were performed prior to our *in vivo* experiments to determine the most consistently effective method of bacterial recovery. In the end, bacterial recovery from both materials was found to be greatest after vortexing the material in 0.9% saline. For these studies, two independent experiments demonstrated that the mean percent recovery of MRSA was 98.9% for ADM and 98.6% for SIS.

For the *in vivo* experiments, the explanted biologic mesh was submerged in 1 mL of sterile saline. The sample was then vortexed to dissociate adherent bacteria, and serial dilutions were plated on LB agar. After twenty-four hours bacterial counts were performed in triplicate. If bacteria grew from the cultured sample, they were scored as positive.

### Biomechanical Analysis

The biomechanical properties of each type of biologic mesh were determined by ultimate tensile strength, and modulus of elasticity before and after inoculation with MRSA. These properties are expressed by most biological tissues when a load is applied. These properties were determined immediately after graft explantation. Ultimate tensile strength (i.e. stress) was defined as the maximum force per cross sectional area that is applied to a material. Modulus of elasticity (an indicator of stiffness) was defined as the stiffness or ability to resist deformation (i.e. the tendency of a material to undergo elastic deformation when a force is applied; increase modulus of elasticity  = increase stiffness of the material). It is equal to stress versus strain ratio, so an increase in deformation would lead to a decrease in the modulus if the stress were held constant.

The mechanical properties were measured using an electromechanical testing system (MTS Systems Corporation, Eden Prairie, MN) equipped with the ReNew upgrade package 1122 (MTS Systems Corporation, Eden Prairie, MN) and an Instron 1,000 lb load cell (Instron, Norwood, MA). System control and data analysis was accomplished at a sampling rate of 60 Hz with TestWorks 4 software (MTS Systems Corporation, Eden Prairie, MN) in displacement control mode. For each sample, uniaxial strain was applied at a rate of 30 mm/min until failure was detected. Failure was defined as a reduction in applied load of eighty percent of the maximum load. During elongation, force/displacement data was collected and the ultimate tensile strength (peak stress; MPa), strain at break (mm/mm) and Modulus of elasticity (MPa) was recorded for each sample. Once failure was detected, as described above, the test was concluded.

### Histologic analysis

Six sections, five micrometers thick, were cut from each sample, stained with Hematoxylin and Eosin (H&E), and examined using light microscopy. Each slide was assessed and subjectively graded by a pathologist blinded to the treatment group for the following characteristics: inflammation, depth of inflammatory response, neovascularization and cellular re-population response. Histological grading was performed as shown in Table 1. The inflammatory response is represented by polymorphonuclear leukocytes per high power field for acute inflammation. Other inflammatory cells (macrophages, lymphocytes, eosinophils) were not counted. For the depth of the inflammatory response the specimen cross-section (thickness) was divided into three parts and the depth of inflammation was scored as one-third, two-thirds or full-thickness. Neovascularization was characterized by the presence of capillaries containing red blood cells growing into the graft. Cellular re-population was defined as a re-population of acellular collagenous membrane by nuclei of fibroblasts. (The depth of revitalization was assessed in a similar manner to the depth of inflammatory response). Histopathologic findings for each group were recorded, and the findings in each group 10 and 20 days after implantation were compared.

**Table 1:** Histology Scoring.

	Host Response	Score
Inflammation	0–4 PMNs/HPF	1
	5–20 PMNs/HPF	2
	>20 PMNs/HPF	3
	Diffuse band-like infiltrate (diffuse to numerous to count)	4
Depth of Inflammation	Inflammatory cells not present	1
	Inflammatory cells present within one-third of tissue matrix	2
	Inflammatory cells present within two-third of tissue matrix	3
	Inflammatory cells present within entire tissue matrix (full-thickness)	4
Neo-Vascularization	No or rare capillaries	1
	Few capillaries (<5 capillaries/HPF)	2
	Many capillaries (5–10 capillaries/HPF)	3
	Abundant capillaries present (granulation tissue)	4
Cellular re-population	Tissue matrix containing no nuclei of fibroblasts	1
	Tissue matrix containing nuclei of fibroblasts within one-third of matrix	2
	Tissue matrix containing nuclei of fibroblasts within two-third of matrix	3
	Tissue matrix containing nuclei of fibroblasts within full thickness of matrix	4

PMNs, polymorphonuclear cells; HPF, high power field;

*Cellular re-population of the acellular collagenous matrix by cellular collagen containing nuclei of fibroblast.

Note. 40 x magnifications.

### Statistical analysis

Data represent the mean ± SEM. Data was analyzed using GraphPad InStat (ver. 3.0; Oberlin Drive, San Diego, CA USA). For continuous variables, three or more group comparisons were analyzed using a one-way analysis of variance with Bonferroni post-test as indicated in the text. Comparison of two groups were done using Mann-Whitney test. For categorical values, Fisher's exact tests were used, and a *p* value <0.05 was considered significant.

## Results

### Macroscopic examination

All animals had a normal post-operative recovery and none died during the study period. No animals exhibited any drainage from or dehiscence of the surgical wounds or mesh extrusion from the skin. Upon opening the pocket, all implanted materials could be located and separated from its surrounding tissue with minimal dissection. We did not observe any abscesses containing significant amounts of white, pus-like material within the implant pocket or surrounding the implant on gross observation. Some erythema of the pocket tissue was present in the experimental groups but not in control (non-infected) animals. In only 1 of the SIS implants (10^9^ MRSA; 20 days) it was noted to be delaminated and tore upon removal. This animal was excluded from the experimental results. A small fluid collection in the implant area, consistent with seroma/hematoma, was noted in six animals (ADM 3, SIS 3). The control implants had minimal surrounding tissue adhered to their surface. This tissue could simply be peeled away from the implants.

### Bacterial Recovery from Explanted Mesh and Bloodstream infection rates

A comparison of the rates of mesh colonization is presented in table 2. Overall, in the experimental groups, 81% of the implanted mesh material had recoverable bacteria upon explantation. There were no significant differences in the implant colonization rates at the end of each time point (table 2). Although we observed a mesh colonization rate of 58% (7/12) for ADM and 92% (11/12) for SIS at the lower inoculum of MRSA (10^5^), this was not statistically significant. In contrast, at the higher inoculum (10^9^ cfu MRSA) the ADM group demonstrated 83% mesh colonization compared to 92% for the SIS implants (table 2). Systemic infection signs were not observed in any of the experiment groups. Bacterial cultures from the blood stream of the rats did not show growth in any of the groups.

**Table 2:** Methicillin Resistant *S. aureus* Recovery from Explanted Biologic Mesh.

Study Group		ADM	SIS	
Inoculum size (MRSA)	Day post inoculation	No. Colonized/total (%)	No. Colonized/total (%)	p value
10^5^	10	3/6 (50)	5/6 (83)	0.54
10^5^	20	4/6 (67)	6/6 (100)	0.45
10^9^	10	5/6 (83)	6/6 (100)	1.0
10^9^	20	5/6 (83)	5/6 (83)	1.0

**Figure 1:**
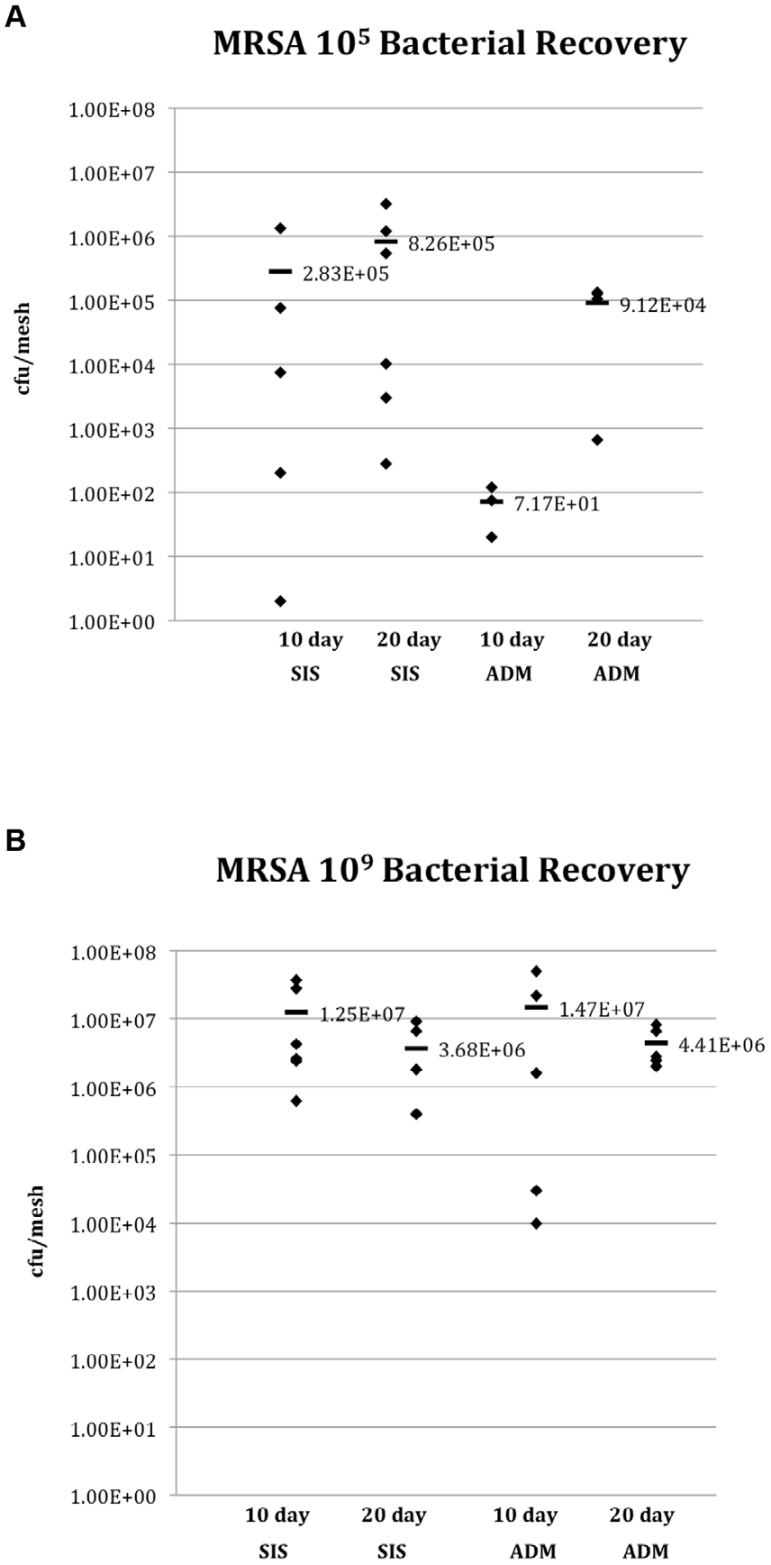
Bacterial recovery from the mesh at explanted at 10 and 20 days post-inoculation with 10^5^ (A) and 10^9^ (B) cfu MRSA.

The quantities of *Staphylococcal* bacteria (colony forming units/mesh) recovered from the different groups are shown in figures 1A and B. Both biologic meshes sustained significant bacterial presence throughout the implantation period with mean bacterial recovery counts slightly less than or greater than the original inoculums. In contrast to SIS, few bacteria were recovered from ADM after exposure to 10^5^ MRSA for 10 days. However, as the inoculation levels and time post inoculation increased, the bacterial recovery increased. None of the control implants had recoverable, viable *Staphylococcal* bacteria recovered from the biologic mesh.

### Biomechanical Properties

**Figure 2:**
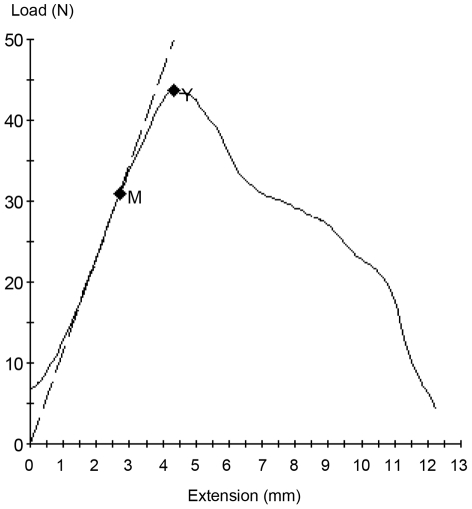
A representative stress-strain curve obtained from material testing. The dashed line indicates the linear region of the curve, the slope of which is the modulus of elasticity. The point labeled M is the proportional limit that corresponds to the end of the linear region of the curve and correlates with the transition from elastic deformation to plastic deformation. During elastic deformation, if the load were removed the material would return to its original size and with no permanent deformation. Once the material progresses to plastic deformation it has undergone permanent deformation and removing the load will no longer return the material to its original size. The point labeled Y is the ultimate tensile strength, which indicates the maximum load applied to the material.

A representative stress-strain curve for the bioprosthesis is shown in Figure 2. The initial slope of these curves represents the modulus of elasticity, and the peak represents the ultimate tensile strength.

#### Ultimate Tensile Strength

**Figure 3:**
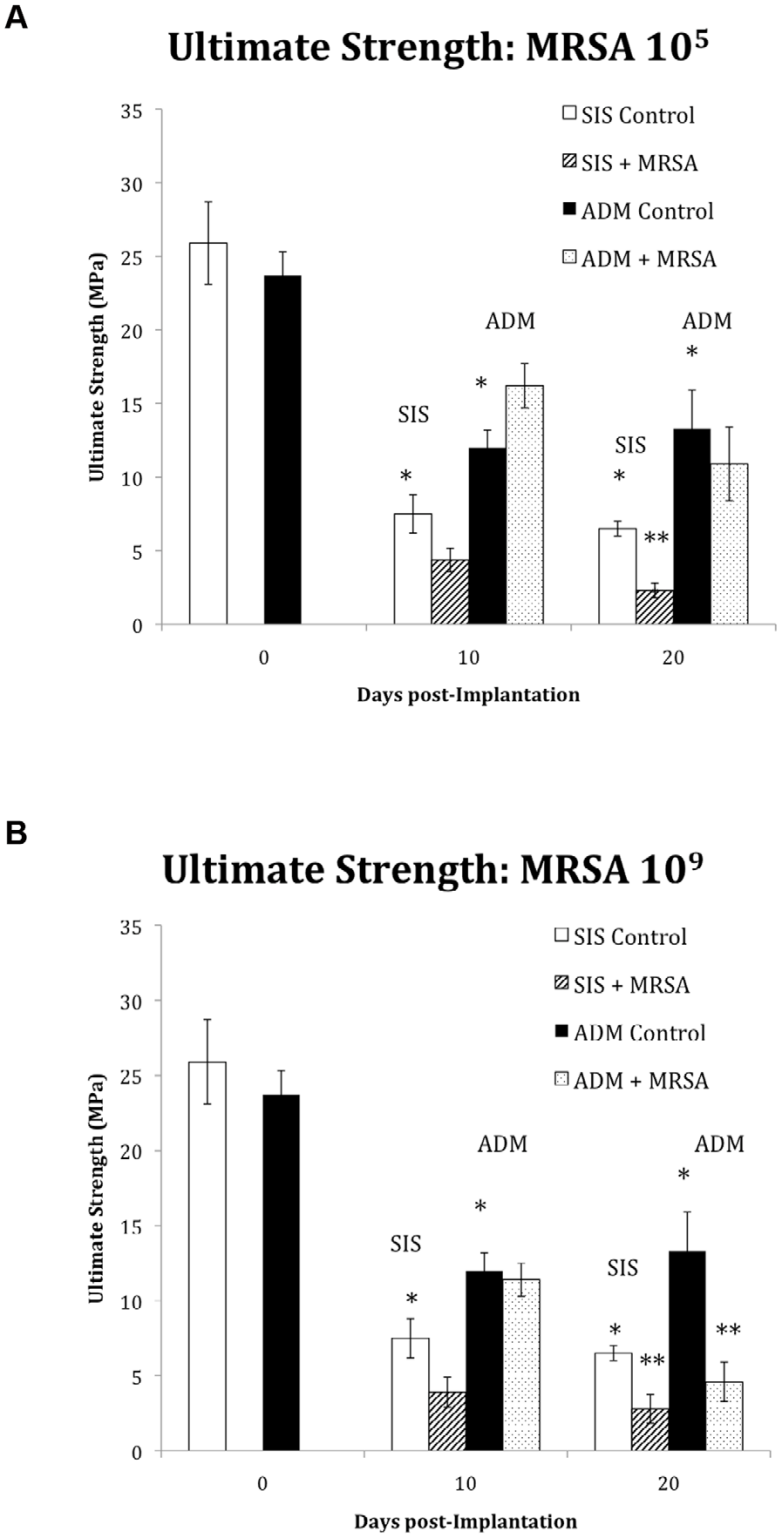
Ultimate strength (MPa) of SIS and ADM at 0, 10, and 20 days in response to an inoculation with 10^5^ (A) and 10^9^ (B) cfu MRSA. White bars (SIS) and Black bars (ADM) represent the control (non-inoculated) values for the 2 biologic meshes at the different time points. Both materials exhibited the greatest reduction in ultimate strength at 20 days post inoculation with 10^9^ MRSA. * Indicates a statistically significant difference between the control groups. ** Indicates a statistically significant difference between inoculated and control groups. ANOVA

There was no significant difference in the ultimate tensile strength between the un-implanted (day 0) ADM (23.7±1.6 MPa) and SIS (25.9±2.8 MPa). Following implantation, the control mesh experienced a significant time dependent decrease in ultimate tensile strength (figure 3 A&B). The mean ultimate tensile strength of the control ADM 10 and 20 days after implantation was significantly greater than that of control SIS (p = 0.05 and 0.03 respectively).

In the bacterial contamination groups, exposure to MRSA appeared to further weaken the materials compared to controls. In addition, significant material differences were observed in response to this bacterial encounter. As shown in figure 3A, at 20 days post inoculation, the ultimate tensile strength of ADM was reduced by only 18% in response to 10^5^ (p>0.05 vs. control, n = 6), but SIS showed a 65% decrease in strength (p<0.01 vs. control, n = 6) at this time point. When a higher inoculum was used (10^9^ cfu MRSA), the ultimate tensile strength of both SIS and ADM were significantly reduced compared to controls at 20 days post inoculation (p<0.05; Figure 3B).

Significant differences in material properties also emerged when colonized ADM was compared with colonized SIS at both 10 and 20 days. Indeed, the following generalizations were noted regarding the mechanical performance of the materials tested in response to 10^5^ cfu MRSA: ultimate strength at 10 days ADM> SIS(p = 0.002), at 20 days ADM>SIS (p = 0.008), in response to 10^9^ cfu MRSA: ultimate strength at 10 days ADM> SIS (p = 0.005), at 20 days ADM = SIS (p = 0.15).

#### Modulus of Elasticity

**Figure 4:**
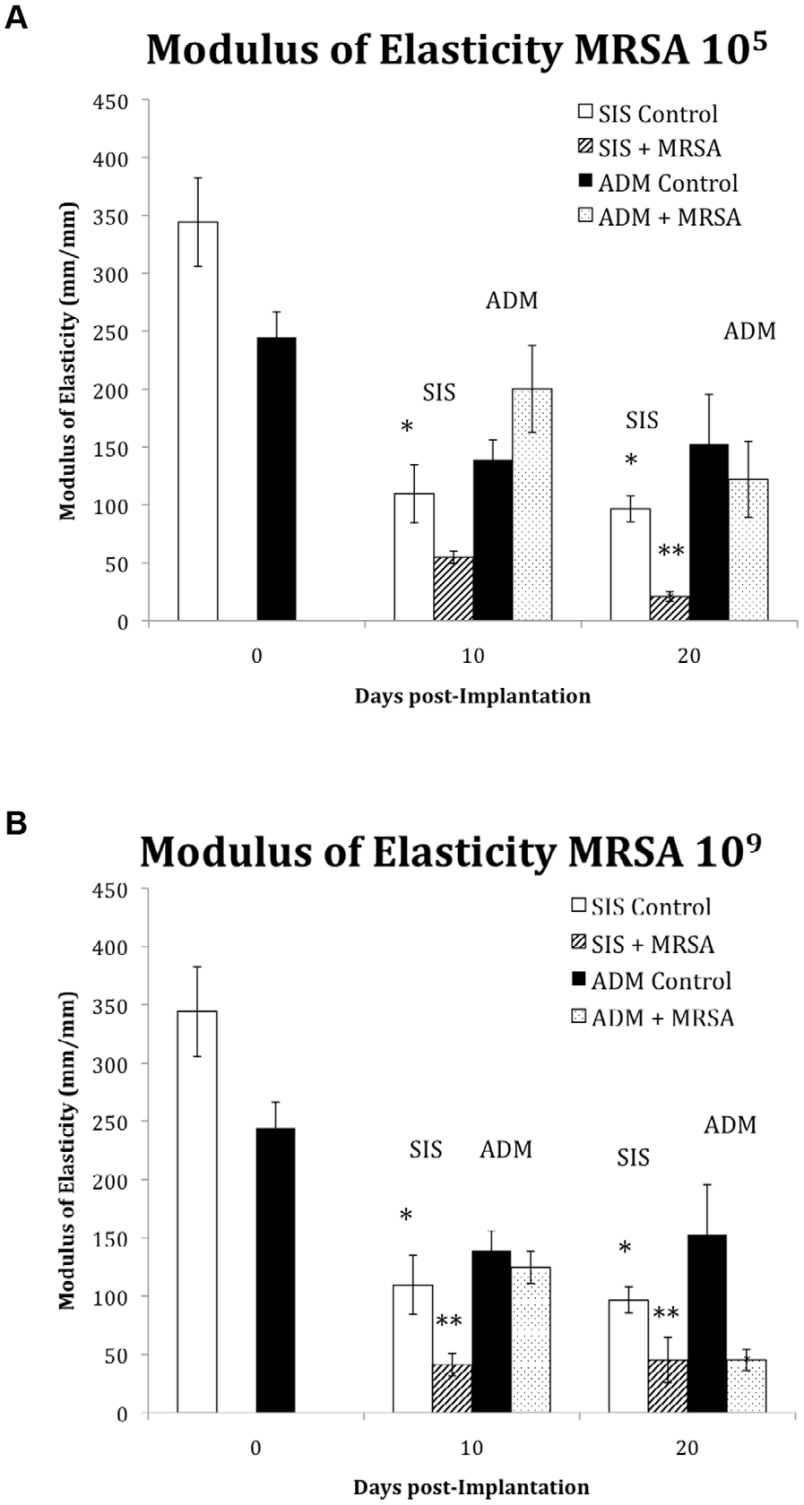
The modulus of elasticity (MPa), a measure of the stiffness of a given material, was used to evaluate the elastic modulus of the biologic meshes at 0, 10 days and 20 days in response to an inoculation with 10^5^ (A) and 10^9^ (B) cfu MRSA. White bars (SIS) and Black bars (ADM) represent the control (non-inoculated) values for the 2 biologic meshes at the different time points. SIS showed the earliest changes in the modulus of elasticity. * Indicates a statistically significant difference between the control groups. ** Indicates a statistically significant difference between inoculated and control groups. ANOVA.

The modulus of elasticity of the un-implanted (day 0) ADM (244.7±22.7 MPa) was slightly lower than SIS (344.2±38.3 MPa) however this was not statistically different (p>0.05). After implantation, control mesh experienced a decrease in the modulus of elasticity (Figure 4 A&B). The corresponding modulus of elasticity of control ADM and SIS at 10 days was 139.1±17.1 and 109.6±25.1 MPA, respectively.

In the bacterial contamination groups, after inoculation with MRSA (10^5^), the modulus of elasticity did not significantly change compared to control values at 10 days for the ADM or SIS. However as shown in figure 4A, at twenty days post-infection, the modulus of elasticity of SIS decreased by 78% in response to inoculation with 10^5^ MRSA (n = 6, p<0.01 vs. control) while the ADM demonstrated only a 10% decrease (p>0.05 vs. control). When the inoculum dose was increased to 10^9^ MRSA, SIS showed a 78% reduction in the modulus of elasticity at 10 days (p<0.05 vs. control) but ADM showed only a 5% reduction (p>0.05 vs. control). However, at 20-day post inoculation with 10^9^ MRSA, the SIS showed no further reduction while ADM showed a 70% decrease in the modulus of elasticity (figure 4B).

When colonized ADM was compared with colonized SIS the following generalizations were observed regarding the modulus of elasticity of the materials in response to 10^5^ cfu MRSA: 10 days ADM> SIS (p = 0.002), at 20 days ADM>SIS (p = 0.004); in response to 10^9^ cfu MRSA: modulus of elasticity at 10 days ADM> SIS (p = 0.002), at 20 days ADM = SIS (p = 0.24).

### Histology

The results of the histological analysis of the meshes are presented in table 3. The random samples were evaluated for acute inflammation, neo-vascularization, and cellular re-population. The histologic evaluation of the samples generally showed variable findings between the ADM and SIS implants. Although more pronounced in the SIS controls, mild inflammation was evident at 10 days in both control (non-infected) biologic mesh materials. As time passed, the degree of inflammation in the control meshes decreased. In contrast to ADM, the SIS control implants showed very little neo-vascularization and cellular re-population by day 10. On the 20^th^ day, there was an increase in the proportion of blood vessels and cellular re-population compared to the 10^th^ day in both control implants.

**Table 3:** Mean Histological Scores.

	ADM					
	10 Day			20 Day		
	Control	MRSA 10^5^	MRSA 10^9^	Control	MRSA 10^5^	MRSA 10^9^
Inflammation	2	1	3	1.7	1.3	3.2
Depth of Inflammation	2	2	3	3	3.5	3.4
Neo-vascularization	2	2	3.2*	3	2.5	3.4
Cellular re-population	2	3	3	2.7	2.8	2.6

	SIS					
	10 Day			20 Day		
	Control	MRSA 10^5^	MRSA 10^9^	Control	MRSA 10^5^	MRSA 10^9^
Inflammation	3.5	4	4	1.8	3*	3.5*
Depth of Inflammation	2.3	2.5	2.5	2.2	2	2.8*
Neo-vascularization	1	1.3	1.5	2.2	1.2*	2.2
Cellular re-population	1	1.3	1	2.3	1.4*	2

*p value ≤0.05 vs. control.

After inoculation with MRSA, the degree and depth of polymorphonucleocyte (PMN) infiltration increased indicating a prominent inflammatory response. This inflammatory response however was related to the inoculum size and mesh material. For example as shown in table 3, an intense inflammatory response was noted in the SIS mesh inoculated with 10^5^ MRSA at 10 and 20 days post inoculation, however only mild inflammatory response was noted in the ADM mesh in response to this bacterial encounter. In contrast, both biologic mesh materials exhibited an intense inflammatory response when inoculated with 10^9^ MRSA, at 10 and 20 days post-inoculation.

All implants ultimately induced neo-vascularization. However compared to SIS, newly formed vessels were easily seen within the ADM after just 10 days. In fact, by day 10 the number of new vessels markedly increased in the ADM mesh exposed to an inoculum of 10^9^ MRSA compared to controls (Table 3). This is in stark contrast to the SIS implants at this time point, which exhibited no change in the number of new vessels within the implant after MRSA inoculation. Interestingly by day 20, a fewer number of new vessels were noted in the SIS mesh exposed to an inoculum of 10^5^ MRSA compared to controls.

## Discussion

A contaminated or infected surgical site is considered a relative contraindication for the use of synthetic mesh material employed to repair abdominal wall defects. As a result, many abdominal wall defects are routinely being repaired with biologic prosthetics. Biologic meshes provide a collagen-rich scaffold that allows cellular in-growth and tissue remodeling, thereby setting the stage for an intact hernia repair [6]. Over the last few years, an increasing diversity of these biomaterials, structural designs, preservation types and cross-linking have become available [5]. However, infection and colonization still continues to be problematic for these biologic meshes resulting in implant failure [8], [10]. Preventing infection of these meshes is particularly challenging, especially when the surgery is typically performed in contaminated surgical fields or in patients at high risk of infection. With the expanding use of these surgically implanted biologic meshes, coupled with increased reports of mesh-associated infections, it is relevant to focus on how bacterial infection/colonization affects these biomaterial specifically their biomechanical properties and whether a difference in their structural designs affects their ultimate response to a bacterial encounter.

Colonization or adherence of bacteria on the surfaces of a mesh is a prerequisite for mesh-related infection. In our study *Staphylococcus aureus,* one of the most commonly involved pathogens in infections of prosthetic meshes, was used to colonize the biologic mesh. Moreover, the effects of staphylococcal colonization were assessed for two biologic meshes, each composed of different source materials, structural designs and preservation methods. Our experimental design included a variety of conditions in order to quantify the effect of variations in the duration and dose of the bacterial encounter.

First of all, our data confirm the work by others, which reported that biologic meshes are susceptible to bacterial colonization/infection [13]. In our study, we were able to create a consistent, nonlethal model of biologic mesh colonization *in vivo* achieving a colonization rate of 81%. Interestingly, the ability to clear the initial bacterial load, varied at our lower bacterial inoculums between the different materials as evidenced by our quantitative microbiology results. In our study when compared to SIS, ADM biologic material appeared to initially clear the low dose MRSA inoculation (10^5^) more effectively, with about 40% of them actually not growing any *S. aureus* on quantitative bacterial cultures at 10 days post inoculation. Consequently, the SIS implants had higher bacterial burden (cfu/mesh) at this time compared to ADM. This outcome might have been related to the differences in tissue source, processing, and sterilization techniques during manufacturing. This ability of ADM to clear a bacterial challenge was also demonstrated by other investigators in various animal models [14], [15]. However, over time and with higher inoculums, both meshes in our model became colonized and had significant bacterial recovery on quantitative cultures at explanation. This suggests that with high enough bacterial challenge no mesh could hope to overcome such an overwhelming bacterial encounter.

Beside infection, inflammation has the potential to progressively destroy the structural integrity of these biologic materials [16]–[18]. After implantation both control materials induced a mild inflammatory response that appeared to subside after 20 days, confirming the low immunogenicity reported by others [19], [20]. After implantation and bacterial contamination, the degree of inflammation increased in both materials. Interestingly, the amount and depth of inflammation was typically higher in SIS after inoculation with bacteria compared to ADM especially at low bacteria doses (10^5^). Indeed, inocluated SIS implants caused an immediate and vigorous inflammatory response, with a faster and more marked inflammatory response at an earlier stage compared to ADM. Importantly, the degree of inflammation appeared to correlate with bacterial recovery and the changes in material strength. The extent to which the inflammatory response contributed to the degradation of the materials is unclear from our model, but it cannot be ruled out as a contributing factor.

Any implanted biologic mesh most likely relies on vascular in-growth before it acquires any antimicrobial defense to bacteria. Indeed, if neovascularization is inhibited it may significantly hamper both the immune response to the infection as well as the efficacy of intravenous antibiotic use. In our study we observed visible vascular growth as early as 10 days post implantation. These results were similar with that of other investigators [21], [22]. Although MRSA did not significantly inhibit neovascularization of ADM, inoculation of SIS with MRSA (10^5^) appeared to hinder new vessel formation compared to controls at 20 days post-inoculation.

The biomechanical properties (i.e. strength and stiffness) of any material used to repair abdominal wall defects are important in maintaining the structural integrity of the repair. After implantation both biologic meshes lost a significant amount of strength in the absence of bacterial encounter (controls). In fact, a 71% decrease in the strength of control SIS was observed during the first 10 days after implantation in our rat model. By comparison, at this time point, ADM showed a 49% reduction in strength. Other investigators have reported similar findings in animal models early after implantation [23]. However over time, these meshes have been shown to re-gain their original strength as they become re-populated with more cells (i.e. remodeled) [23], [24]. Despite this, the clinical utility of such a degradable biomaterial ultimately depends on a delicate balance between the rate of degradation and the rate of remodeling. If a mesh degrades prior to adequate cellular infiltration, differentiation, collagen deposition and neo-vascularization the overall quality and strength of the newly formed tissue will be insufficient for abdominal wall repair. In our study, we have shown that the level of contamination can negatively influence the rate of degradation of biologic mesh. Consequently, the biomechanical properties of these materials may be completely different when used in cases of a chronic clinical infection by Staphylococcus aureus [25] as compared to their use in cases of gross contamination, open bowel or even peritonitis [26].

As hypothesized, the performance profile of the biologic mesh varied in response to a bacterial encounter. In the group that received ADM, the ultimate tensile strength, was markedly higher than those in the SIS group. Whereas ADM requires both a higher dose, and a longer time period before showing any signs of significant degradation, SIS begins to exhibit signs of degradation sooner than ADM and with much lower doses. Not only did we observe a decrease in material strength in response to a bacterial encounter but also its modulus of elasticity. The decrease in modulus of elasticity was due to an increase in the strain and a decrease in the stress. Both SIS and ADM exhibited a marked decreased modulus of elasticity when inoculated with high dose MRSA (10^9^) after 20 days. This indicates that in addition to a reduction in overall strength, the materials are exhibiting an increased deformation prior to failure. This increase in deformation could be a mechanism of failure for the materials without the ultimate strength needing to be reduced to physiological levels. An increase in the deformation of the material could lead to recurrent hernia formation without the material failing. These results are in line with the scattered data from clinical reports of bulging after implantation in humans [27].

It has been reported that the mean intra-abdominal pressure while standing is 2.7 kPa and 14.3 kPa when coughing [28]. While the strengths of the colonized biologic mesh in our study materials were still well in excess of physiological pressures exerted in the body, the drastic reduction in strength over as short of a period of time begs the question of how long before they become significant. In order for a catastrophic failure of the material to occur, the decrease in ultimate strength would have to continue to decline. However, deformation of the materials could also render them useless in maintaining the abdominal wall integrity after repair long before a complete failure occurs.

The findings of the present study indicate that our *in vivo* model is a simple and reproducible experimental model to study the various consequences of an bacterial encounter on biologic meshes used for hernia repairs. Such an study should be mandatory for all meshes before they are used for abdominal wall hernia repair. Overall, we observed that biologic meshes become colonized with bacteria and this colonization results in a reduction in the materials biomechanical properties in a time, dose and material dependent manner. Thus, with time and after a considerable bacterial encounter, the biologic meshes, are not only deforming more readily, but also that they are become weaker, in that they fail at a lower ultimate tensile strength.

Infection or colonization of any implant is difficult if not almost impossible to overcome and represent a formidable clinical challenge. The following experimental study highlights some of the concerns with biologic mesh when placed in an infected field. Specifically, we urge caution when considering biologic mesh in heavily contaminated environments as this can lead to implant failures. With this understanding, we believe that steps need to be taken to safeguard these materials from bacterial colonization. Incorporation of antimicrobial agents, biofilm modifications and bacterial interference agents into devices themselves ought to be further investigated. Newer products and modifications to exiting products may further enhance the benefits of biologic mesh particularly in challenging cases.
